# Reduction of *Coxiella burnetii* Prevalence by Vaccination of Goats and Sheep, the Netherlands

**DOI:** 10.3201/eid1703.101157

**Published:** 2011-03

**Authors:** Lenny Hogerwerf, René van den Brom, Hendrik I.J. Roest, Annemarie Bouma, Piet Vellema, Maarten Pieterse, Daan Dercksen, Mirjam Nielen

**Affiliations:** Author affiliations: Utrecht University, Utrecht, the Netherlands (L. Hogerwerf, A. Bouma, M. Pieterse, M. Nielen);; Animal Health Service, Deventer, the Netherlands (R. van den Brom, P. Vellema, D. Dercksen);; Central Veterinary Institute of Wageningen UR, Lelystad, the Netherlands (H.I.J. Roest)

**Keywords:** Q fever, Coxiella burnetii, bacterial vaccine, bacteria, bacterial shedding, goats, sheep, the Netherlands, expedited, research

## Abstract

Recently, the number of human Q fever cases in the Netherlands increased dramatically. In response to this increase, dairy goats and dairy sheep were vaccinated against *Coxiella burnetii*. All pregnant dairy goats and dairy sheep in herds positive for Q fever were culled. We identified the effect of vaccination on bacterial shedding by small ruminants. On the day of culling, samples of uterine fluid, vaginal mucus, and milk were obtained from 957 pregnant animals in 13 herds. Prevalence and bacterial load were reduced in vaccinated animals compared with unvaccinated animals. These effects were most pronounced in animals during their first pregnancy. Results indicate that vaccination may reduce bacterial load in the environment and human exposure to *C*. *burnetii*.

Q fever, which is caused by *Coxiella burnetii*, is a worldwide zoonotic infectious disease, and ruminants are the main reservoir for human infections (*1*–*3*). Ruminant infections may occasionally result in abortions, which are associated with shedding of large amounts of bacteria in placentas and birth fluids (*4*). Human infections have been reported mainly in persons handling infected animals and their products (*5*–*8*). However, this disease has not been perceived as a major public health risk for the general population. In 2007, a major epidemic occurred in the general population in the Netherlands (*9*), which resulted in >2,300 reported cases in 2009. An explanation for the emergence of human Q fever was abortion clusters in goat herds beginning in 2005 within an intensified dairy goat production system (*10*–*15*). This hypothesis was substantiated by epidemiologic studies, which indicated a possible spatial link between dairy goat farms and human cases (*16*).

Reduction of the number of human cases was considered essential by public health authorities in the Netherlands. One of the intervention measures taken was vaccination of dairy goats against *C. burnetii* (*17*). This measure assumed that vaccination would reduce abortions and bacterial shedding to levels that would reduce the number of human cases in the following year. Vaccination began in 2008 and intensified in 2009. As the number of cases of *C. burnetii* infection in patients doubled in 2009, policymakers applied a precautionary principle and decided to cull all pregnant dairy goats or sheep on infected farms before the 2010 kidding season. This measure was implemented at the end of 2009 and thereby precluded any field analysis of vaccine efficacy in the spring of 2010. However, there was an opportunity to sample animals shortly after they were humanely killed. The purpose of this study was to quantify the effect of vaccination on bacterial load in excreta of pregnant animals.

## Materials and Methods

### Q Fever in the Netherlands since 2005

**Figure 1:**
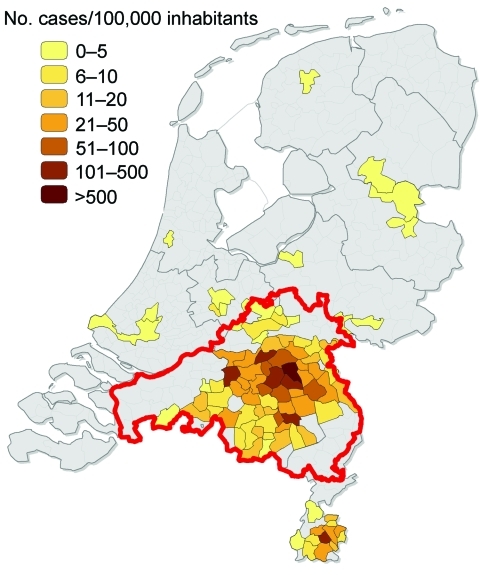
Density of 1,133 reported cases of acute Q fever in humans per municipality, the Netherlands, January 1–June 10, 2009. Area outlined in red is where vaccination of dairy goats and sheep was mandatory in 2009 (Noord Brabant Province and parts of adjacent provinces). Data were obtained from the National Institute for Public Health and the Environment, Statistics Netherlands, the Food and Consumer Product Safety Authority, and the Ministry of Agriculture, Nature and Food Quality.

Human Q fever cases in the Netherlands increased from 168 in 2007 to 1,000 in 2008 and 2,355 in 2009, mainly in Noord-Brabant Province (*11*). A campaign of voluntary vaccination of dairy goats began at the end of 2008 in the area of the 2007 human case cluster and was followed by mandatory vaccination of all dairy goat and dairy sheep on farms with >50 animals in a larger area in 2009. This vaccination zone included Noord-Brabant Province and parts of adjacent provinces because the supply of vaccine was not sufficient for all small ruminant farms in the Netherlands and because most human cases had occurred in that area (Figure 1) (*13*).

Additional control measures implemented in the fall of 2009 were a bulk milk test every 2 weeks to detect *C. burnetii*–infected herds and to monitor *C. burnetii*–negative herds, movement and breeding bans for dairy goats or sheep, and culling of all pregnant dairy goats or sheep on infected farms. Health authorities considered a farm to be infected when 2 consecutive bulk milk samples were positive by PCR, as tested by 2 laboratories, including the national reference laboratory (*17*). Thus, culling included pregnant goats in vaccinated herds and pregnant goats in unvaccinated herds located outside the vaccination zone. Culling was conducted from the end of December 2009 through May 2010.

### Vaccine

The vaccine used was Coxevac (Ceva Santé Animale, Libourne, France). This vaccine was not registered in the Netherlands at the time of the study, but authorities had issued a temporary exemption. The vaccine is a phase I vaccine containing inactivated *C. burnetii* strain Nine Mile (*18*). It was recommended that uninfected animals be vaccinated twice over a 1-month interval before pregnancy. Although efficacy in dairy goats was not shown, the expected effects in vaccinated animals were reduced infection, abortion, and bacterial shedding if animals were infected after vaccination (*19*–*21*).

### Study Design

For various reasons related to regulations of the national culling operation, unvaccinated dairy goats from 5 farms, vaccinated dairy goats from 7 farms, and unvaccinated dairy sheep from 1 farm were included in this study. Farms were not randomly selected but were selected on the basis of convenience of culling date, vaccination status, and agreement of farmers to participate in the study. We sampled 100 animals per farm, 50 pregnant and lactating animals (old animals), and 50 nulliparous animals (young animals). With this sample size, we expected to be able to detect a 20% difference in *C. burnetii* prevalence between vaccinated and unvaccinated animals and between old and young animals. We tested 3 types of samples: 1) uterine fluid, to detect animals with a high risk for shedding around parturition; 2) vaginal mucus, to be consistent with test results of other studies (*19*–*21*); and 3) milk, because herds were monitored on the basis of results of bulk milk tests.

On the day before culling, animals were selected and marked on the farm by the study team; authorities identified pregnancies by using sonography. We selected pregnant animals that were closest to giving birth because it was expected that these animals had the highest number of *C. burnetii* in birth fluids, which would facilitate detection of infection (*4*). After animals were humanely killed on farms, marked animals were transported in a separate container to a rendering plant (Rendac BV, Son, the Netherlands), where they were unloaded onto a concrete floor and prepared for sampling.

### Sampling

Uterine fluid was obtained by using a 9-mL monovette EDTA blood collection system (Sarstedt, Nümbrecht, Germany) and a Bovivet 2.10 mm × 60 mm needle (Terumo Europe NV, Leuven, Belgium). Before obtaining uterine fluid, we made an incision in the linea alba cranial from the udder, moved part of the uterus to an extraabdominal position, and cleaned the uterus with alcohol-soaked cotton balls. We also cleaned the vulva with alcohol-soaked cotton balls and then obtained a swab sample from the vagina wall by using a dry and sterile cotton-tipped Cultiplast swab (LP Italiana SPA, Milan, Italy). These 2 samples were obtained from all selected animals. Additionally, from old animals we obtained a milk sample, which was collected into a 30-mL sterile tube. The teat was cleaned with alcohol-soaked cotton balls before sampling, and the first few streams of milk were discarded. All samples were frozen at −40°C within a few hours after sampling and were sent to the laboratory to be analyzed after the end of the culling period.

### Diagnostic Test

Quantitative real-time PCR was performed for all samples. Milk samples were analyzed at the Animal Health Service by using the Taqvet *Coxiella burnetii* TaqMan Quantitative PCR (Laboratoire Service International, Lissieu, France). Swabs and uterine samples were analyzed by the national reference laboratory by using an in-house real-time PCR specific for the *C. burnetii* insertion sequence 1111a gene (*22*). Results for the 3 sample types were given as positive, negative, or doubtful on the basis of cycle threshold (C_t_) values, in which a value <36.01 was considered positive and a value >40 was considered negative. A negative result indicated that no specific signal was detected in a maximum of 40 cycles. Values between 36.01 and 40 were reported as doubtful on the basis of <100% reproducibility. For additional analysis, we considered all samples with C_t_ <40 as positive.

### Statistical Analyses

Vaccine efficacy was calculated for young and old animals separately for all 3 sample types according to the following equation: [% (positive test result, unvaccinated) – % (positive test result, vaccinated)] / [% (positive test result, unvaccinated)] (*23*). This efficacy can be interpreted as the percentage of positive samples (C_t_ <40) prevented by vaccination in a vaccinated population.

Influence of vaccination and parity on test results of individual animals was examined by using logistic regression (*24*) for the 3 sample types. We included vaccination status and parity group in the model as explanatory variables. Herd was included as a random factor to incorporate the fact that observations within a herd are dependent in the model. For uterine samples and vaginal swabs, we used the equation logit (fraction of positive test results) = parity (old) + vaccination status stratified by parity (young or old vaccinated) + random herd effect stratified by vaccination status (vaccinated or unvaccinated herds). For milk samples, we used the equation logit (fraction of positive test results) = vaccination status (vaccinated) + random herd effect stratified by vaccination. Vaccine effect was quantified by calculating the odds ratio (OR).

For positive samples only, we tested whether vaccination had an effect on the relative amount of bacteria present in each sample type, as indicated by the C_t_ value. A C_t_ value closer to 0 indicates a higher bacterial concentration in the sample relative to a C_t_ value closer to 40. We performed survival analysis on samples with C_t_ values for which the C_t_ value at which a sample result becomes positive is considered the event. Hazard ratio (HR) indicates the rate at which samples from unvaccinated animals become positive compared with samples from vaccinated animals (*25*). No correction for herd level was necessary and no correction for parity was possible because of the low number of bacterial shedders per group. For each of the 3 samples types, we used the equation C_t_ value (of positive samples only) = vaccination status (vaccinated).

Kaplan-Meier curves were plotted to show bacterial load in samples from old vaccinated, young vaccinated, old unvaccinated, and young unvaccinated animals. Statistical analyses were performed by using R software (*26*). For logistic regression, the function glmer() in lme4 in R software (*27*) was used. For survival analysis, the functions Surv() and coxph() in Survival in R software (*28*) were used. The model fit of all models was assessed by using the likelihood ratio test.

## Results

### Background Information for Individual Farms

Information for each farm is shown in Table 1. Three farms (B, F, and K) did not have a history of animals with Q fever before the end of 2009 when their bulk milk PCR results changed from negative to positive during the monitoring period, which suggested a recent infection. Abortion caused by Q fever had been diagnosed in 2008 on sheep farm X. On all other farms, >1 bulk milk ELISA or PCR results were positive for *C.* *burnetii* in 2008 or 2009. Animals in vaccinated herds were supposedly vaccinated twice in 2009, with the exception of farm M, where the first vaccination was given after abortions had occurred.

**Table 1:** Characteristics of goat and sheep farms sampled for *Coxiella burnetii*, the Netherlands, January–April 2010*

Farm	No. animals culled	No. live animals	Vaccination period	Bulk milk sample PCR result and date of change, 2010†
Unvaccinated goats				
A	550	178	NA	+
B	102	530	NA	Mar
F	53	938	NA	Mar
K	121	649	NA	Feb
L	324	367	NA	+
Unvaccinated sheep				
X	128	378	NA	Jan
Vaccinated goats				
H	365	673	2009 Aug–Dec	Jan
M	719	3,557	2009 Dec–2010 Jan	+
P	625	1,750	2009 Sep–Dec	+
Q	685	281	2009 Aug–Oct	+
R	3,595	0	2009 Sep–Oct	+
S	180	358	2009 Oct	+
T	1,081	83	2009 Apr–Sep	+

*Data from Animal Health Service and the Food and Consumer Product Safety Authority. Animals were vaccinated with Coxevac (Ceva Santé Animale, Libourne, France). No. live animals is the number of nonpregnant animals remaining after culling. NA, not applicable; +, positive. †Shown are farms that had a positive PCR result at the start of the culling period (+) and those for which a PCR result changed from negative to positive during the culling period (date).

### Effect of Vaccination on Bacterial Shedding

**Figure 2:**
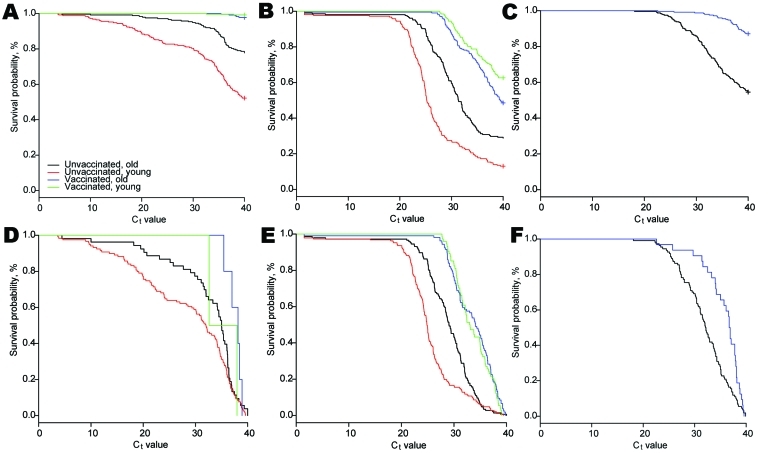
Kaplan-Meier curves for cycle threshold (C_t_) values of all samples (A–C) and for samples with positive and doubtful results for *Coxiella burnetii* (C_t_ <40) (D–F), the Netherlands, January 1–June 10, 2009. A, D) Uterine fluid; B, E) vaginal mucus; C, F) milk. Old, pregnant and lactating; young, nulliparous.

Crude test results are summarized in Table 2. The percentage of *C. burnetii*–positive animals on each farm is shown in Figure 2. For vaccinated animals, 0.43% of uterine samples, 30% of vaginal swabs, and 4% of milk samples were positive (C_t_ <36.01). For unvaccinated animals, 26% of uterine samples, 76% of vaginal swabs, and 33% of milk samples were positive. Prevalences within vaccinated herds and unvaccinated herds varied substantially (Table 2).

**Table 2:** Quantitative PCR results and prevalence for samples positive for *Coxiella burnetii* for 957 animals in 13 small ruminant herds, the Netherlands, January–April 2010*

Farm	Group	Uterine fluid					Vaginal mucus					Milk			
		No.	Pos	D	% (95% CI)		No.	Pos	D	% (95% CI)		No.	Pos	D	% (95% CI)
Unvaccinated goats															
A	Young	46	0	0	0 (0–6)		0	0	0	NA		0	0	0	NA
	Old	47	0	2	0 (0–6)		0	0	0	NA		52	8	2	15 (6–25)
B	Young	74	35	16	47 (36–59)		76	75	0	99 (96–100)		0	0	0	NA
	Old	26	10	2	39 (20–57)		26	26	0	100		26	17	8	65 (47–84)
F	Young	49	35	4	71 (59–84)		53	52	0	98 (95–100)		0	0	0	NA
	Old	0	0	0	NA		0	0	0	NA		0	0	0	NA
K	Young	26	17	5	65 (47–84)		32	32	0	100		0	0	0	NA
	Old	28	12	3	43 (25–61)		39	39	0	100		34	33	0	97 (91–100)
L	Young	37	0	0	0 (0–8)		37	2	9	5 (0–13)		0	0	0	NA
	Old	58	0	0	0 (0–5)		58	1	3	2 (0–5)		51	2	3	4 (0–9)
Unvaccinated sheep															
X	Young	17	5	2	29 (8–51)		17	17	0	100		0	0	0	NA
	Old	79	11	13	14 (6–22)		82	76	1	93 (87–98)		79	19	18	24 (15–34)
Vaccinated goats															
H	Young	48	1	0	2 (0–6)		49	1	5	2 (0–6)		0	0	0	NA
	Old	50	0	0	0 (0–6)		50	6	11	12 (3–21)		37	0	0	0 (0–8)
M	Young	50	0	1	0 (0–6)		49	46	2	94 (87–100)		0	0	0	NA
	Old	47	1	3	2 (0–6)		48	47	0	98 (94–100)		47	5	12	11 (2–20)
P	Young	0	0	0	NA		0	0	0	NA		0	0	0	NA
	Old	0	0	0	NA		30	12	9	40 (23–58)		30	1	0	3 (0–10)
Q	Young	49	0	0	0 (0–6)		50	2	8	4 (0–9)		0	0	0	NA
	Old	49	0	1	0 (0–6)		50	2	12	4 (0–9)		50	0	2	0 (0–6)
R	Young	0	0	0	NA		0	0	0	NA		0	0	0	NA
	Old	10	0	0	0 (0–26)		0	0	0	NA		10	0	0	0 (0–26)
S	Young	46	0	0	0 (0–6)		50	4	6	8 (0–16)		0	0	0	NA
	Old	25	0	0	0 (0–11)		28	2	5	7 (0–17)		28	1	5	4 (0–10)
T	Young	49	0	0	0 (0–6)		0	0	0	NA		0	0	0	NA
	Old	47	0	0	0 (0–6)		0	0	0	NA		46	3	3	7 (0–14)

*No., no. tested; pos, no. with positive result; D, no. with doubtful result; %, prevalence; CI, confidence interval; young, nulliparous; NA, not applicable; old, pregnant and lactating.

Vaccine efficacy for uterine sample results was 98% for young animals and 90% for old animals. Vaginal sample vaccine efficacy was much lower (57% and 28%) for young and old animals, respectively. Vaccine efficacy for milk sample test results was 72% (Table 3). All logistic regression model fits and survival model fits were better than those of null models according to likelihood ratio tests.

**Table 3:** Efficacy of vaccination against *Coxiella burnetti* for 957 animals in 13 small ruminant herds, the Netherlands, January–April 2010*

Group	Uterine fluid					Vaginal mucus					Milk			
	No.	Pos	D	E, %		No.	Pos	D	E, %		No.	Pos	D	E, %
Unvaccinated														
Young	249	92	27	NA		215	178	9	NA		NA	NA	NA	NA
Old	238	33	20	NA		205	142	4	NA		242	79	31	NA
Subtotal	487	125	47	NA		420	320	13	NA		242	79	31	NA
Vaccinated														
Young	241	1	1	98		198	53	21	57		NA	NA	NA	NA
Old	228	1	4	90		206	69	37	28		248	10	22	72
Subtotal	470	2	5	NA		404	122	58	NA		248	10	22	NA
Total	957	127	52	NA		824	442	71	NA		490	89	53	NA

*No., no tested; pos, no. with positive result; D, no. doubtful; E, vaccine efficacy; young, nulliparous; NA, not applicable; old, pregnant and lactating.

For vaccinated animals, uterine samples from young animals were 0.5% as likely to be positive for *C. burnetii* (OR 0.005, 95% CI 0.0002–0.1200), and uterine samples from old animals were 3.2% as likely to be positive (OR 0.032, 95% CI 0.002–0.580) than samples from unvaccinated young animals. For unvaccinated animals, old animals were 44% as likely to be positive than young animals (OR 0.44, 95% CI 0.25–0.78) (Table 4). Results from the vaginal swabs were comparable; vaccinated young animals were 1.5% as likely to be positive for *C. burnetii* than unvaccinated young animals (OR 0.015, 95% CI 0.0006–0.3500). Milk from vaccinated old animals was 4% as likely to be positive for *C. burnetii* than milk from unvaccinated old animals (OR 0.04, 95% CI 0.003–0.380) (Table 5).

**Table 4:** Multivariate logistic regression of prevalence of *Coxiella burnetii* in culled animals from 13 small ruminant herds, the Netherlands, January–April 2010*

Group	Uterine fluid			Vaginal mucus	
	OR (95% CI)	p value		OR (95% CI)	p value
Unvaccinated					
Young	1	NA		1	NA
Old	0.44 (0.25–0.78)	<0.05		0.22 (0.08–0.64)	<0.05
Vaccinated					
Young	0.005 (0.0002–0.12)	<0.05		0.015 (0.0006–0.35)	<0.05
Old	0.03 (0.002–0.58)	<0.05		0.13 (0.006–3.01)	0.2

*A random herd effect was included. OR, odds ratio; CI, confidence interval; young, nulliparous; NA, not applicable; old, pregnant and lactating.

**Table 5:** Univariate logistic regression of prevalence of *Coxiella burnetii* in milk samples from culled animals in 13 small ruminant herds, the Netherlands, January–April 2010*

Group	OR (95% CI)	p value
Old, unvaccinated	1	NA
Old, vaccinated	0.04 (0.003–0.38)	<0.05

*A random herd effect was included. OR, odds ratio; CI, confidence interval; old, pregnant and lactating; NA, not applicable.

### Effect of Vaccination on C_t_ Value

In uterine fluid, vaccinated animals had an HR that was half that of unvaccinated animals (HR 0.49, 95% CI 0.34–0.70), which indicated that unvaccinated *C. burnetii*–positive animals had higher relative amounts of bacteria on the basis of C_t_ value. This effect was similar for vaginal mucus (HR 0.34, 95% CI 0.28–0.42) and milk (HR 0.54, 95% CI 0.39–0.75) (Table 6).

**Table 6:** Univariate survival analysis of PCR Ct values for Coxiella burnetii in positive and doubtful samples from culled animals in 13 small ruminant herds, the Netherlands, January–April 2010*

Group	Uterine fluid			Vaginal mucus			Milk	
HR (95% CI)	p value	HR (95% CI)	p value	HR (95% CI)	p value
Unvaccinated	1	NA		1	NA		1	NA
Vaccinated	0.49 (0.39–0.70)	<0.05		0.34 (0.28–0.42)	<0.05		0.54 (0.39–0.75)	<0.05

*C_t_, cycle threshold; HR, hazard ratio; CI, confidence interval; NA, not applicable.

C_t_ values for uterine fluid and vaginal mucus were lowest for *C. burnetii*–positive, unvaccinated young animals, which suggested that they had the highest relative amount of bacteria (Figure 2). C_t_ values were similar in bacteria-positive vaccinated animals, regardless of parity group, which indicated lower but similar shedding levels in all vaccinated animals. For milk samples, C_t_ values were lower for unvaccinated animals than for vaccinated animals.

## Discussion

This study showed that vaccination of dairy goats against Q fever with Coxevac reduced the percentage of animals in which bacteria were detected and bacterial load in uterine fluid, vaginal swabs, and milk. Reduced prevalence was most prominent in uterine fluid and in young animals. Because shedding of bacteria may be quantitatively highest during parturition, abortion, and subsequent periods, these results suggest that vaccination may reduce environmental contamination, thereby contributing to reduction of risk for human exposure and associated human cases of Q fever.

Our findings are consistent with those of other studies. In a clinical trial of cattle, Guatteo et al. (*20*) demonstrated that vaccine was effective in reducing the probability of becoming a bacterial shedder when given to uninfected animals before pregnancy. Arricau-Bouvery et al. (*21*) showed that vaccination of 17 goats in a clinical trial decreased excretion of *C. burnetii*. Rousset et al. (*19*) conducted a field study of a goat herd infected with *C*. *burnetii* and found that vaccination did not prevent shedding but did reduce bacterial load in vaginal swabs of primiparous animals.

Although these studies provided useful data on the effect of vaccination, these data were based on a limited number of observations. The advantages of our study were that it was based on a larger number of field samples (957 animals from 13 herds) obtained from animals vaccinated under field conditions and that it tested uterine fluid, which is likely to be a good proxy for shedding at the time of kidding. A disadvantage of our study was its observational nature, in which vaccination was not conducted randomly at the herd, animal, parity, or infection levels, as would have been conducted in a clinical trial.

In unvaccinated herds *C. burnetii* was detected more often in uterine fluid of young animals than in old animals. However, no parity difference was observed for vaccinated herds. Rousset et al. (*19*) observed a reduced bacterial load in vaginal swabs in primiparous goats only. We also observed that the bacterial load was most reduced in young vaccinated animals. However, vaccinated young and old animals had similar bacterial loads in uterine fluid and vaginal mucus (Figure 2). Our results suggest that vaccination is more protective in nulliparous animals than in parous animals. Further investigations are required to determine whether the association between vaccination and bacterial shedding depends on vaccination before a first or subsequent pregnancy or on vaccination before or after natural exposure, and to elucidate underlying mechanisms.

As reported by Guatteo et al. (*20*), the time of vaccination before or during breeding may affect its effectiveness. In our study, whether all animals had been vaccinated before breeding was not known. On 1 farm, all animals were vaccinated after breeding, and most vaccinated animals with a positive test result for *C. burnetii* came from this farm. When we excluded this farm from the analyses, we observed a stronger effect of vaccination, which indicated that the effect of vaccination could have been underestimated.

However, the efficacy of vaccination may also have been overestimated. With exception of the dairy sheep farm, all unvaccinated herds with a high prevalence of *C.* *burnetii*–positive uterine samples had no known history of Q fever until milk PCR results became positive during the culling period. This result suggested a recent introduction of the infectious agent. In other unvaccinated herds that had only a few positive uterine samples, *C. burnetii* was circulating before the culling period. All vaccinated herds appeared to have histories of *C. burnetii* infection. This factor makes it difficult to conclude whether absence of positive uterine samples in vaccinated herds was caused by vaccination or was a combined effect of vaccination and an immune response after natural infection.

Another study limitation is that the stage of pregnancy can affect the amount of *C. burnetii*; bacterial load in secreta may increase sharply during the last stage of pregnancy (*4*). Although we attempted to select animals that were closest to giving birth, not all animals sampled were in the same stage of pregnancy, and the average duration of pregnancy may have differed from farm to farm. Because data about gestation stage were lacking, we did not include this factor in our analyses.

Goats and sheep in the Netherlands were vaccinated to reduce the number of human cases of Q fever. However, other countries use a different strategy. In Australia, persons at risk are vaccinated against Q fever (*29*). In France, cattle are vaccinated to prevent economic losses caused by abortions (*30*). No substantial numbers of human cases of Q fever have been reported in these countries (*31*). The effect of vaccination in the Netherlands on reduction of human exposure could not be quantified. However, the low number (≈350) of human cases in 2010 compared with those in 2009 (*32*) suggests a beneficial effect of intervention measures. The relationship between bacterial shedding, environmental contamination, and human cases needs further investigation.

Our results showed that in uterine fluid, vaginal mucus, and milk, *C. burnetii* prevalence and load were reduced in vaccinated animals in the Netherlands. These effects were most pronounced in young primiparous animals. We can reasonably assume that vaccination under field conditions contributed to reduction of shedding of *C. burnetii* by dairy goats and dairy sheep, which in turn may contribute to reduction of the risk for human exposure.
